# Association between polymorphic CAG repeat lengths in the androgen receptor gene and susceptibility to prostate cancer: A systematic review and meta-analysis

**DOI:** 10.1097/MD.0000000000007258

**Published:** 2017-06-23

**Authors:** Zhiqiang Qin, Xiao Li, Peng Han, Yuxiao Zheng, Hanyu Liu, Jingyuan Tang, Chengdi Yang, Jianzhong Zhang, Kunpeng Wang, Xiaokang Qi, Min Tang, Wei Wang, Wei Zhang

**Affiliations:** a Department of Urology, The First Affiliated Hospital of Nanjing Medical University; b Department of Urologic Surgery, The affiliated Cancer Hospital of Jiangsu Province of Nanjing Medical University; c First Clinical Medical College of Nanjing Medical University, Nanjing; d Department of Urology, The First People's Hospital of Lianyungang City, Lianyungang; e Department of Urology, Subei People's Hospital, Yangzhou, China.

**Keywords:** androgen receptor, CAG repeat polymorphisms, meta-analysis, prostate cancer

## Abstract

**Background:**

Previous studies have been conducted to reveal the relationship between androgen receptor CAG polymorphism and risk of prostate cancer, yet the results were elusive and controversial. Thus, this meta-analysis was performed to clarify this association.

**Methods:**

To obtain the relevant available studies, online databases PubMed, Embase, and Web of science were searched until September 1st, 2016. The pooled odds ratios (ORs) with 95% confidence intervals (CIs) were used to assess the strength of such association. Subgroup analyses were conducted based on ethnicity and source of controls. Moreover, Begg's funnel plots and Egger's linear regression test were conducted to test the publication bias.

**Results:**

Overall, our results enrolled 51 studies indicated that significant increased risk of prostate cancer was associated with androgen receptor CAG polymorphism (OR  =  0.77, 95% CI: 0.67–0.89). In addition, compared with CAG repeat <20, 22, carriers of ≧20, 22 repeats had decreased risk of prostate cancer (cut-off point  =  20: OR  =  0.27, 95% CI: 0.13–0.52; cut-off point  =  22: OR  =  0.82, 95% CI: 0.70–0.97). However, when cut-off point  =  23, no significant result was detected in such association (pooled OR  =  0.88, 95% CI: 0.63–1.24). When cut-off point is 22, the results were positive only in Asian population (OR  =  0.53, 95% CI: 0.32–0.89) in the subgroup analysis by ethnicity. Besides, when the studies were stratified by source of controls, the results were not significant in both the subgroup of population-based controls and hospital-based controls.

**Conclusions:**

This meta-analysis suggested the carriers of short polymorphic CAG repeats might increase susceptibility to prostate cancer, which held potential as a detecting marker of the risk of prostate cancer.

## 1 Introduction

Prostate cancer (PCa) is one of the most common malignant tumor in men all around the world. Only after lung cancer, PCa is considered the second leading cause of cancer-related deaths among men in USA in 2016.^[1,2]^ Many potential risk factors, including cigarettes, eating patterns, age, endocrine system, environment and genetic factors, might influence the complicated etiology of PCa.^[3–7]^ Although the accurate pathogenic mechanism of PCa remains no fully clear, it has been testified that genetic polymorphisms seem to play an essential role in sporadic cases of PCa.^[8]^

The androgen receptor, a ligand-dependent transcriptional regulator, induces the actions of testosterone and dihydrotestosterone. Eight exons constitute the androgen receptor gene, which is located on X chromosome (q11-q12). Moreover, there are 2 polymorphic trinucleotide repeats in exon 1 of the AR gene that encode poly-glutamine (CAG)n.^[9]^ Androgens are of great significance in the occurrence and progression of PCa, whose function is realized via the androgen receptor.^[4]^ Previous studies have observed that CAG repeat length differed in different populations, and it was inversely connected to the AR gene transcription activity, which could meditate the AR's reaction to androgens. Eventually, the CAG repeat length was related to the occurrence and evolution of PCa.^[10,11]^ Furthermore, multiple epidemiological studies reported the correlation between the CAG repeats and risk and aggression of PCa.^[12]^

Subsequently, numerous previous studies have shown the relevance between CAG repeat length and the risk and progression of PCa. Nonetheless, the consequences of these researches remained inconsistent or even contradictory, and some disputable presumptions existed. Therefore, all qualified studies were included in the meta-analysis to provide statistical evidence and estimate the real relationship between CAG repeats and PCa risk.

## 2 Materials and methods

Online databases including PubMed, Embase, and Web of science were searched thoroughly for relevant studies about the association of CAG repeat length and PCa risk, with the last search update on September 1st, 2016. We used the combination of the following keywords: (“androgen receptor CAG” or “CAG repeat polymorphism”), (“polymorphism” or “variants”), (“prostate cancer” or “prostatic carcinoma”). In addition, we brought in eligible literature via hand-searching from reference of original studies and reviews. If studies had partly overlapped subjects, only these with latest or largest sample size were included.

Involved studies had to meet the inclusion criteria as follows: (1) a case-control design was used; (2) evaluation of the association between CAG repeat polymorphisms and PCa risk; (3) sufficient data provided to calculate the odds ratios (ORs) and the corresponding 95% confidence intervals (CIs). Besides, the major exclusion criteria were as follows: (1) no available CAG repeat length data; (2) without control groups; (3) duplicates of previous publication.

### 2.1 Data extraction

Two investigators (QZQ and HP) participated in reviewing the identified researches independently to determine whether each study was eligible for inclusion. The data were drawn from studies involved separately and any disagreement was resolved by a discussion with a third reviewer (LX), according to the main point of view. All the following information were extracted from each study and were registered in a standardized form: year of publication, first author's name, ethnicity, source of controls, detected sample, the number of cases and controls, cut-off point of CAG repeat length, and frequency of CAG repeat polymorphisms in cases and controls, respectively.

### 2.2 Statistical analysis

The pooled ORs with 95% CIs were utilized to evaluate the strength of association between the CAG repeat polymorphisms and PCa susceptibility. A 95% CIs without 1 for OR indicated a meaningfully increased or reduced PCa risk. According to the *P* values of study heterogeneities, the fixed-effects model based on the Mantel-Haenszel method and the random-effects model based on the DerSimonian-Laird method were separately applied to pool the data.^[13]^ If the heterogeneity was detected (*P* < .05 or *I*^2^ >50%), the random-effects model would be more appropriate; otherwise, the fixed-effect model was conducted to perform this meta-analysis. Subsequently, subgroup analysis was also conducted to explore the potential sources of heterogeneity by ethnicity and source of controls. After that, we performed the sensitivity analysis to examine the stability and reliability of the results by calculating the results again by omitting 1 individual study at every turn. Moreover, Begg's funnel plots and Egger's linear regression test were conducted to test the publication bias between the researches.^[14]^*P* values, being all 2-sided, were considered statistically meaningful when less than 0.05.^[15]^ All statistical analyses were carried out with Stata software (version 12.0; StataCorp LP, College Station, TX).

## 3 Results

### 3.1 Studies characteristics

**Figure 1:**
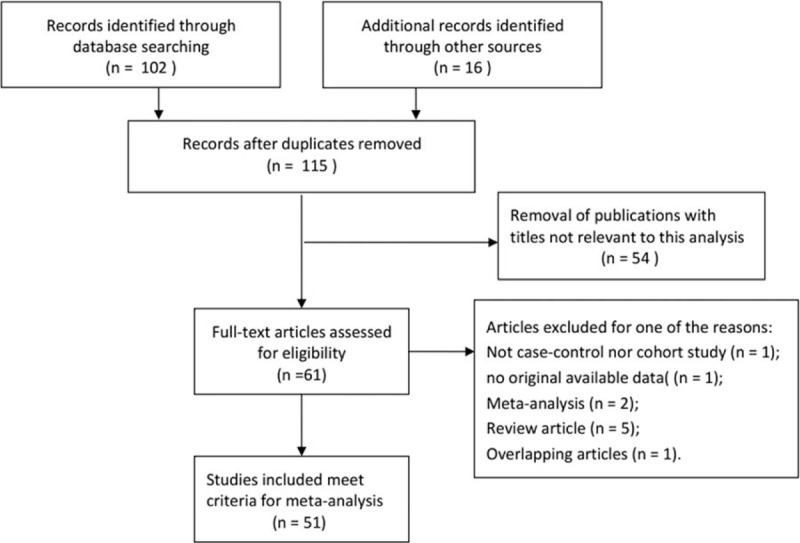
Flow diagram of literature search and selection process.

Based on the inclusion and exclusion criteria, a total of 51 case-control studies including 11,891 cases and 15,351 controls were included in the current meta-analysis, and the details characteristics of all selected studies were listed in Table 1. The flowchart of literature search and selection process was showed in Fig. 1. The sample size of each study ranged between 66 and 2,512. Among these previous studies, there were 5 different ethnic groups, including 24 studies conducted in Caucasians population,^[16–39]^ 12 studies based on Asian population,^[40–51]^ 3 studies from African population,^[52–54]^ 3 studies from Brazilian population,^[55–57]^ and 9 studies from Mixed population.^[58–66]^ Furthermore, in order to distinguish between different sources of controls, we consisted of 27 population-based studies and 17 hospital-based studies. DNA was extracted from whole blood in almost these studies, and only PCR was utilized as the genotyping method.

**Table 1:**
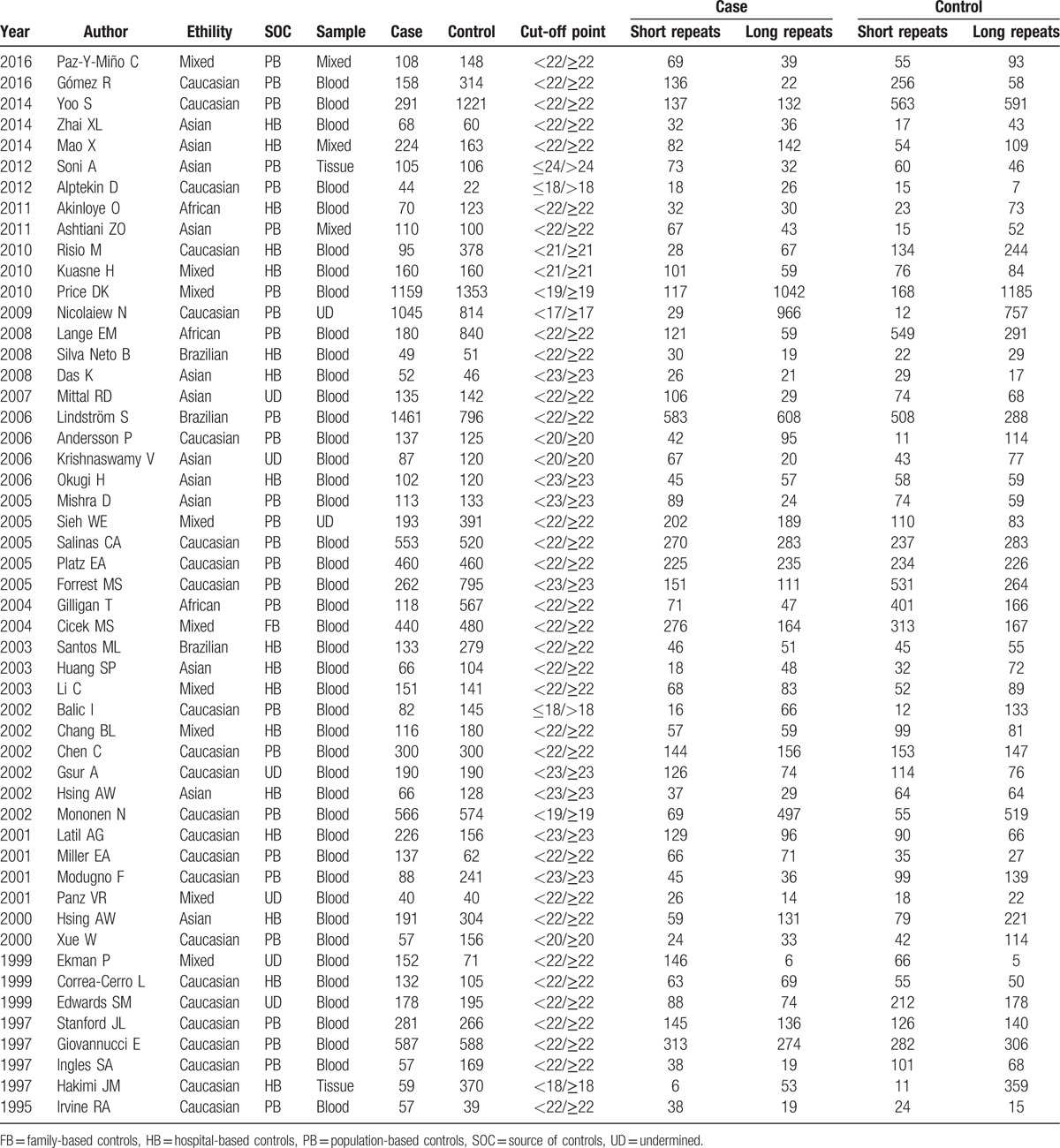
Characteristics of individual studies included in the meta-analysis.

### 3.2 Quantitative synthesis results

**Figure 2:**
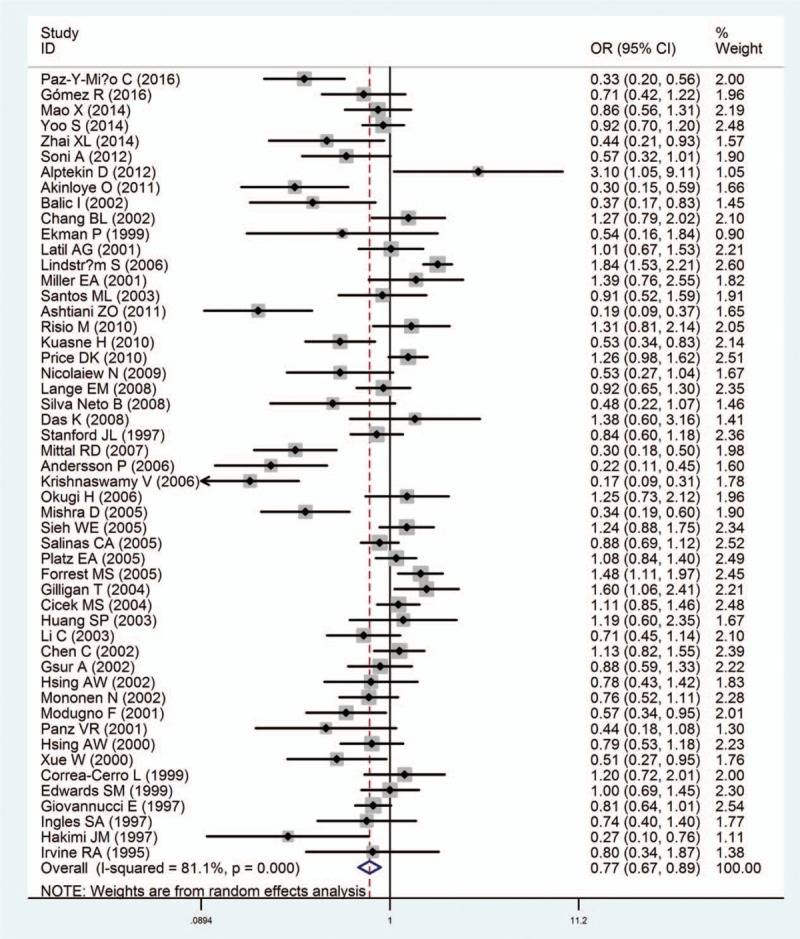
Forest plots of the association between androgen receptor CAG polymorphism and prostate cancer susceptibility.

**Figure 3:**
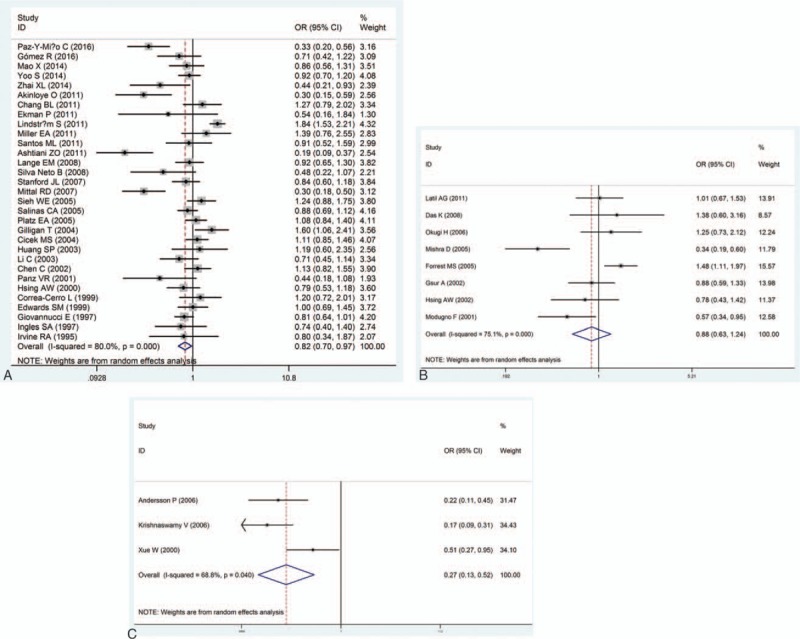
Forest plots of the association between androgen receptor CAG polymorphism and prostate cancer susceptibility. (A) Cut-off point  =  22; (B) cut-off point  =  23; (C) cut-off point  =  20.

The main results of the meta-analysis about the associations between AR gene polymorphisms CAG repeats and the risk of PCa were shown in Table 2. Generally speaking, the pooled OR of the enrolled 51 studies was 0.77 (95% CI: 0.67–0.89) (Fig. 2). Because no high-qualified studies provided the specific distributions of AR CAG repeat counts, we focused on 3 cut-off points to explain such association, including ≥23 repeats of CAG polymorphism versus others, ≥22 repeats versus others and ≥20 repeats versus others. In total, there were 31 reports comparing ≥22 CAG repeats with others, 8 reports comparing ≥23 repeats with others, and 3 reports comparing ≥20 repeats with others. Thus, carriers of ≧20, 22 repeats had decreased risk of PCa in the random-effects model (cut-off point  =  20: OR  =  0.27, 95% CI: 0.13–0.52; cut-off point  =  22: pooled OR  =  0.82, 95% CI: 0.70–0.97) compared with CAG repeat<20, 22. However, when cut-off point  =  23, no significant result was detected in the relationship between AR gene polymorphisms CAG repeats and the risk of PCa (pooled OR  =  0.88, 95% CI: 0.63–1.24) (Fig. 3).

**Table 2:**
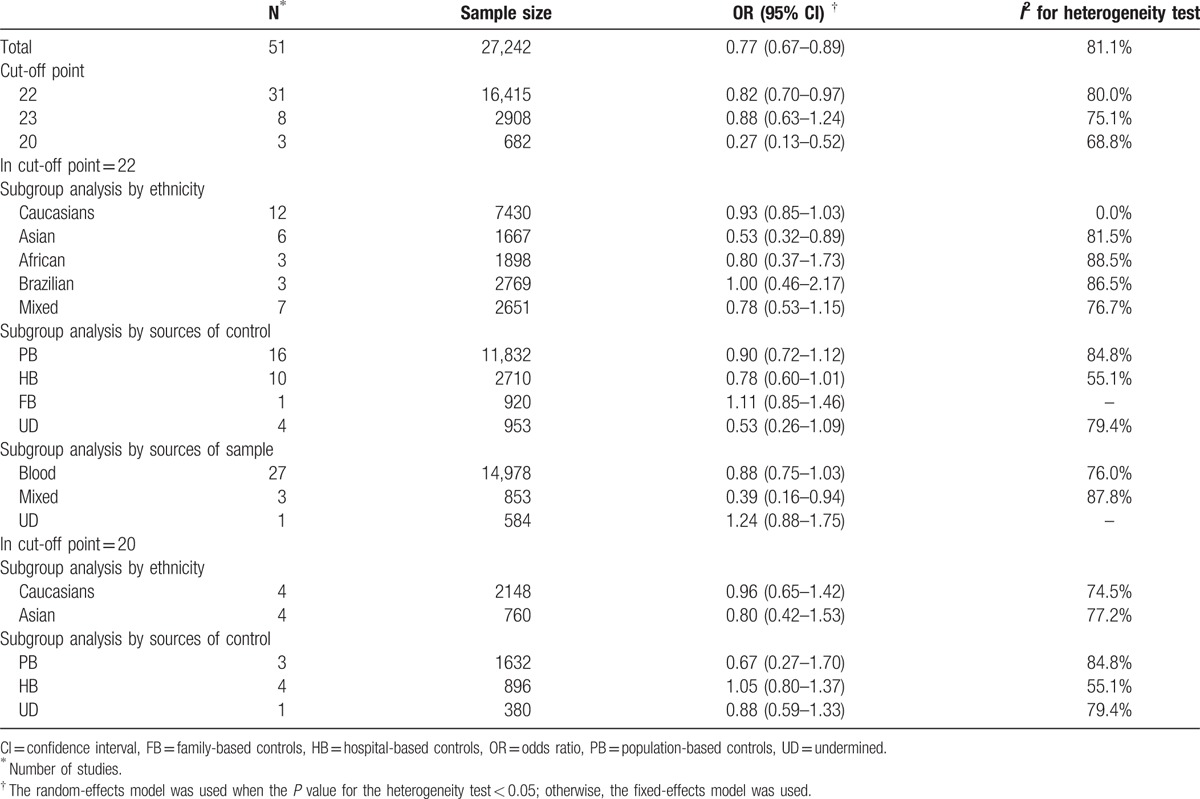
Meta-analysis results of association between androgen receptor CAG polymorphism and prostate cancer risk.

**Figure 4:**
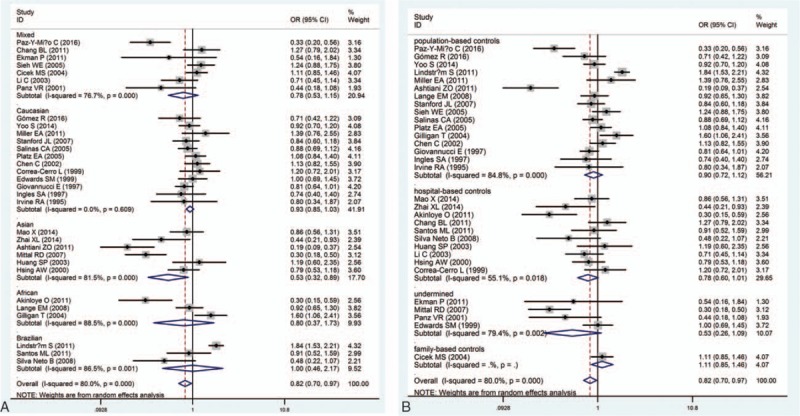
Forest plots of subgroup analysis of the association between androgen receptor CAG polymorphism and prostate cancer susceptibility in the cut-off point of polymorphic CAG repeat lengths  =  22. (A) Stratified by ethnicity; (B) stratified by source of controls.

When cut-off point of polymorphic CAG repeat lengths was 22, the results were positive only in Asian population (pooled OR  =  0.53, 95% CI: 0.32–0.89) in the subgroup analysis by ethnicity (Fig. 4A). Besides, when the studies were stratified by source of controls, the results was no significant in both the subgroup of population-based controls (pooled OR  =  0.90, 95% CI: 0.72–1.12) and hospital-based controls (pooled OR  =  0.78, 95% CI: 0.60–1.01) (Fig. 4B). As a consequence, for AR gene polymorphism association, the carriers of short CAG repeats held higher PCa risk than those with long CAG repeats, especially in Asian ethnicity.

### 3.3 Sensitivity analysis

While omitting 1 individual study each time, sensitivity analysis was applied to detect the influence of each study on the pooled OR by repeating the meta-analysis. The sensitivity analysis for AR gene polymorphism association of CAG repeat polymorphisms and PCa risk in the overall population showed that no single study affected the pooled ORs significantly.

### 3.4 Publication bias

**Figure 5:**
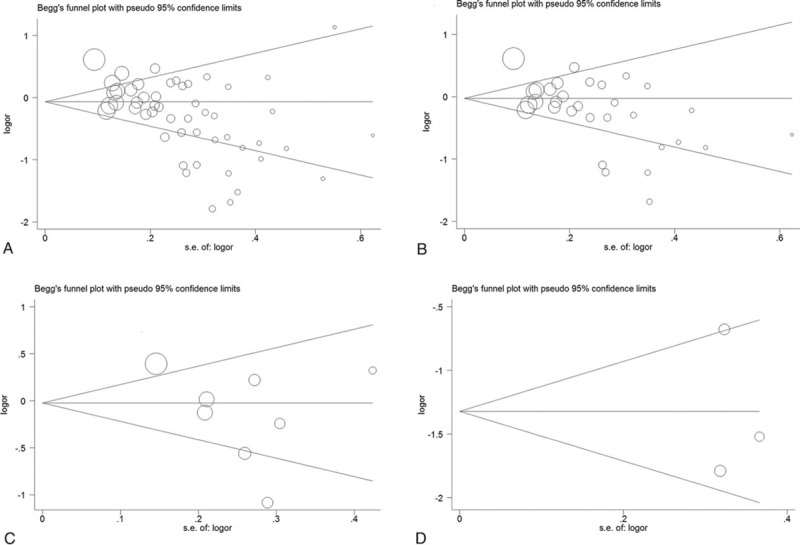
Begg's funnel plot of publication bias test. (A) Total studies; (B) cut-off point  =  22; (C) cut-off point  =  23; (D) cut-off point  =  20.

Begg's funnel plot and Egger's test were used to evaluate the publication bias of the literature. The shapes of the funnel plots seemed obvious evidence of asymmetrical, identifying meaningful publication bias (Begg's test was 0.001; Egger's test was 0.001) (Fig. 5). In addition, publication bias was observed according to different cut-off points. (1) *P*-value was .028 for Begg's test and .003 for Egger's test for the cut-off point  =  22. (2) *P*-value was .458 for Begg's test and .225 for Egger's test for the cut-off point  =  23. (3) *P*-value was .602 for Begg's test and .987 for Egger's test for the cut-off point  =  20.

## 4 Discussion

Meta-analysis is a kind of powerful tool that can provide more credible results than 1 individual study and explicate controversial conclusions. For this reason, we made use of meta-analysis to illustrate the possible relationship between CAG repeats and susceptibility to PCa. A recent meta-analysis suggested that the absolute difference in number of repeats between cases and controls was <1 repeat, although the presence of shorter repeats seemed to be modestly associated with PCa risk.^[67]^ Another meta-analysis showed that AR CAG repeat polymorphism with ≧20 repeats might confer a protective effect among the PCa patients with 45 years older but not all the patients with PCa.^[68]^ However, these results remained unclear. In this meta-analysis, we systematically researched the association between AR CAG repeats polymorphism and PCa susceptibility. Generally speaking, the AR CAG repeats polymorphism was associated with PCa risk, and the shorter CAG repeats polymorphism was more susceptible to PCa.

PCa, as a carcinoma of prostate, is a complex and multifactorial disease which has affected interethnic males. The incidence rate and mortality of PCa in westerners is 10-fold more than that in Chinese.^[1,2]^ Besides, there is also a significantly higher mortality among African Americans than in Caucasians in the USA.^[4,69]^ The approximated newly diagnosed PCa cases have been up to 180,890 and 26,120 mortalities in USA in 2016.^[1]^ Furthermore, the growth of prostate cell is stimulated by androgen via androgen receptor, so AR signaling plays an important role in prostate growth and maintenance. In the progress of cancer, abnormal AR signaling is related to PCa development. Therefore, the pathogenesis of PCa is closely related to androgen whose function is mediated by the androgen receptor.

Since PCa usually occurs at older age, the androgen receptor gene codes for a protein that has an androgen-binding domain, DNA-binding domain and N-terminal domain, which contain 2 polymorphic trinucleotide repeats (CAG and GGC). The prostate is an androgen-dependent organ whose cell cycle is mediated by the interaction between the receptor and androgens. In addition, the AR transcription activity, correlated to the PCa risk, is implicated in polymorphism of CAG repeat length which has been demonstrated in vitro, and the AR with a shorter CAG repeats has greater transcription than that with longer CAG repeats.^[70,71]^ Overall, the normal distribution of the CAG triad nucleotide repetitive sequences is reported in a range of 6 to 39, with an average of 19 to 20 in African–Americans, 21 to 22 in Caucasians, 22 to 23 in Asians, and 23 in Hispanics.^[72]^ Remarkably, the length of CAG repeats was usually longer in Asians than in Caucasians. Therefore, the present meta-analysis aimed to provide a more powerful and reliable conclusion on the relationship between polymorphic CAG repeat lengths and PCa susceptibility.

The result of this meta-analysis indicated the carriers of short CAG repeats held higher PCa risk than those with long CAG repeats, especially in Asian ethnicity. Moreover, compared with CAG repeat <20, 22, carriers of ≧20, 22 repeats had decreased risk of PCa. However, when cut-off point  =  23, no significant result was detected in the relationship between AR gene polymorphisms CAG repeats and PCa. For the cut-off point of polymorphic CAG repeat lengths  =  22, these findings of subgroup analyses based on ethnicity and control source can be explained as follows. After stratified analysis was performed by ethnicity, we found that CAG repeat length was associated with PCa risk only in Asian population but not statistically meaningful in Caucasian, African, African, or Mixed populations. Though the exact mechanism was unclear, it was likely that different ethnic groups with various genetic backgrounds might have differences in genetic drift and natural selection, resulting in different gene polymorphisms risk of developing PCa. In addition, we conducted stratified analysis by source of controls and the result was not detected significant both in population-based and hospital-based populations. In this meta-analysis, the results were in concordance with these hypotheses of previous studies, which needed to further prove that the carriers of short CAG repeats polymorphism played an important role in the susceptibility of PCa.

Notably, this is a meta-analysis to comprehensively illustrate the impact of CAG repeat polymorphism in response to PCa risk. Nevertheless, several limitations should be taken into consideration and also be emphasized. First, certain results, especially those in each stratified analyses, remain to be further validated because of relatively insufficient data, contributing to potentially limiting the statistical power to investigate the real association. Second, no uniform cut-off point of polymorphic CAG repeat lengths was used in previous studies, suggesting that the result of meta-analysis might exist some merits. Thus, additional studies should pay attention to unified standard in subsequent studies to guaranty reliability of our meta-analysis. What is more, no enough data were extracted from all studies to adjust estimates by other PCa covariates, such as age, cigarettes, drinking status, and so on. Thereby, a more high-qualified analysis would have been provided if more detailed data from individual studies were available. In addition, because only 3 studies were included in the cut-off 20 repeats dataset, we cannot exclude the possibility of publication bias. Therefore, more data were required to analysis these results, when it interpreted the results in this meta-analysis. Moreover, as a multi-factorial disease, PCa is closely concerned with environmental backgrounds and the interaction with various genetic factors instead of the influence of any single gene. Thus, more researches about exploring the risk effects of CAG repeats polymorphism in susceptibility to PCa needed to be further validated in the future.

## 5 Conclusion

The results of the present meta-analysis indicated that polymorphic CAG repeat lengths in the androgen receptor gene were significantly associated with susceptibility to PCa. Meanwhile, the carriers of short CAG repeats might be a strong risk factor of PCa, especially in Asian population. More importantly, our findings need to be further validated whether the AR CAG repeats polymorphism might be a potential etiology and detecting marker for the risk of PCa in the future.
